# Warmer and browner waters decrease fish biomass production

**DOI:** 10.1111/gcb.14551

**Published:** 2019-02-10

**Authors:** Renee M. van Dorst, Anna Gårdmark, Richard Svanbäck, Ulrika Beier, Gesa A. Weyhenmeyer, Magnus Huss

**Affiliations:** ^1^ Department of Aquatic Resources, Institute of Coastal Research Swedish University of Agricultural Sciences Öregrund Sweden; ^2^ Department of Aquatic Resources Swedish University of Agricultural Sciences Öregrund Sweden; ^3^ Department of Ecology and Genetics, Animal Ecology, Evolutionary Biology Centre Uppsala University Uppsala Sweden; ^4^ Department of Aquatic Resources, Institute of Freshwater Research Swedish University of Agricultural Sciences Drottningholm Sweden; ^5^ Wageningen Marine Research IJmuiden The Netherlands; ^6^ Department of Ecology and Genetics, Limnology, Evolutionary Biology Centre Uppsala University Uppsala Sweden

**Keywords:** biomass production, browning, climate change, Eurasian perch, fish, individual body growth, lakes, length distribution, ontogeny, warming

## Abstract

Climate change studies have long focused on effects of increasing temperatures, often without considering other simultaneously occurring environmental changes, such as browning of waters. Resolving how the combination of warming and browning of aquatic ecosystems affects fish biomass production is essential for future ecosystem functioning, fisheries, and food security. In this study, we analyzed individual‐ and population‐level fish data from 52 temperate and boreal lakes in Northern Europe, covering large gradients in water temperature and color (absorbance, 420 nm). We show that fish (Eurasian perch, *Perca fluviatilis*) biomass production decreased with both high water temperatures and brown water color, being lowest in warm and brown lakes. However, while both high temperature and brown water decreased fish biomass production, the mechanisms behind the decrease differed: temperature affected the fish biomass production mainly through a decrease in population standing stock biomass, and through shifts in size‐ and age‐distributions toward a higher proportion of young and small individuals in warm lakes; brown water color, on the other hand, mainly influenced fish biomass production through negative effects on individual body growth and length‐at‐age. In addition to these findings, we observed that the effects of temperature and brown water color on individual‐level processes varied over ontogeny. Body growth only responded positively to higher temperatures among young perch, and brown water color had a stronger negative effect on body growth of old than on young individuals. Thus, to better understand and predict future fish biomass production, it is necessary to integrate both individual‐ and population‐level responses and to acknowledge within‐species variation. Our results suggest that global climate change, leading to browner and warmer waters, may negatively affect fish biomass production, and this effect may be stronger than caused by increased temperature or water color alone.

## Graphical abstract

As lakes get warmer and browner, fish biomass production decreases through different pathways. Warming reduces population biomass and induces a shift toward smaller and younger individuals, whereas browning leads to slower body growth. Our study shows that it is necessary to study multiple climate stressors and both individual‐ and population‐level responses to understand and predict shifts in fish biomass production in a changing climate.

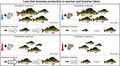

## 1 INTRODUCTION

In northern latitude lakes, warming and darkening of water color caused by elevated concentrations of dissolved organic carbon (DOC) and iron are ongoing processes coupled to climate change, with potentially severe consequences (Dokulil, 2014; Larsen, Andersen, & Hessen, 2011; Monteith et al., 2007; Roulet & Moore, 2006; Solomon et al., 2015; Weyhenmeyer, Müller, Norman, & Tranvik, 2016; Whitehead, Wilby, Battarbee, Kernan, & Wade, 2009). Both warming and darkening of water color, often referred to as browning, have been shown to impact lake ecosystems (Ask et al., 2009; Finstad, Helland, Ugedal, Hesthagen, & Hessen, 2014; Jeppesen et al., 2012; Vasconcelos et al., 2016). Resolving how such changes affect fish biomass production is essential for future ecosystem functioning (Holmlund & Hammer, 1999), fisheries, and food security (FAO, 2016). Studies over small spatial scales suggest that warming and browning could affect fish biomass production (negative effect of decreased light penetration (Karlsson et al., 2009) and DOC in lakes (Karlsson et al., 2015), positive effect of high temperatures in streams (O'Gorman et al., 2016), and see Jeppesen et al. (2010) for an example of the effect of temperature on fish biomass in lakes). Yet, to our knowledge, no study has looked at effects of variation in water temperature and color, alone or in combination, on fish biomass production across a large set of natural systems, acknowledging the potential for additive or interactive effects.

**Figure 1:**
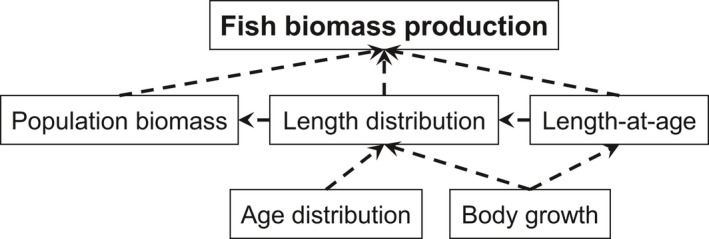
Fish biomass production and its underlying individual‐ and population‐level attributes. Individual body growth underlies changes in length‐at‐age, and together with population age‐distribution (mean age, skewness in age), also changes in population length distributions (mean length, skewness in length), fish biomass, and ultimately fish biomass production. The net effect of warming and browning on fish biomass production may thus depend on changes in interlinked variables responding to warming and browning both at the level of individuals and of populations

To understand variation in fish biomass production across climatic gradients and to make predictions in the face of climate change, we need to recognize that the amount of fish biomass production is mediated by underlying individual‐ and population‐level attributes (Figure 1) and how these respond to variation in temperature and water color. Body growth, which is a fundamental component of individual fitness that influences population‐level responses, is strongly affected by both temperature (Angilletta, Steury, & Sears, 2004) and water color (Benoît, Beisner, & Solomon, 2016; Estlander et al., 2010; Horppila et al., 2010). Depending on the increase in feeding rates relative to the increase in metabolic rates with temperature, warming can have a positive or negative direct effect on body growth (Brown, Gillooly, Allen, Savage, & West, 2004; Lemoine & Burkepile, 2012). In addition, changes in fish body growth in response to warming could emerge via lower trophic level responses (Yvon‐Durocher, Jones, Trimmer, Woodward, & Montoya, 2010). Generally, warming‐induced increases in primary production do not compensate for the increase of metabolic rates of herbivores and predators, often leading to increased top–down control and resource limitation of growth with warming (O'Connor, 2009; Yvon‐Durocher et al., 2012).

In addition to temperature, browning may also affect lake ecosystems and individual body growth of fish in a variety of ways. Browning increases light attenuation in the water column (Jones, 1992; Williamson, Morris, Pace, & Olson, 1999), leading to a decrease in primary production, especially in benthic habitats (Ask et al., 2009; Seekell, Lapierre, Ask et al., 2015; Vasconcelos et al., 2016). This may result in less food resources available for fish, such as zoobenthos (Karlsson et al., 2009). Decreased visibility may also impede foraging ability of visually hunting consumers in lakes, such as fish (Jönsson, Hylander, Ranåker, Nilsson, & Bronmark, 2011; Ranåker, Jönsson, Nilsson, & Brönmark, 2012). Both lower food availability and impeded foraging ability could lead to reduced fish body growth rates. Accordingly, fish body growth has indeed been shown to be lower in a small number of brown lakes than in clear lakes (Estlander et al., 2010; Horppila et al., 2010). In addition, browning may negatively affect fish by reducing the amount of macrophytes, which provide refuge from predators in benthic areas (Solomon et al., 2015). Browning can also reduce fish habitat availability by shifting thermal stratification, leading to a thinner layer of warm surface water high in oxygen on top of a larger cold and potentially deoxygenated area in brown lakes (Solomon et al., 2015). Shading and shifted stratification would predict only negative effects of browning on fish individual body growth. However, increased nutrient availability associated with browning (Sanders et al., 2015) may benefit nutrient‐limited primary producers (especially in the pelagic habitat, Ask et al., 2009; Vasconcelos et al., 2016), which may positively affect the amount of resources available to higher trophic levels. Especially at low levels of browning, these positive ecosystem effects may outweigh those caused by decreased light availability (Finstad et al., 2014; Kelly et al., 2016; Seekell, Lapierre, & Karlsson, 2015). Furthermore, also given the same level of browning, responses may vary among lakes due to differences in lake morphology (Seekell, Byström, & Karlsson, 2018; Solomon et al., 2015). Thus, both increased temperatures and browning may affect fish body growth.

These individual‐level growth responses to warming and browning can vary with body size. In general, small individuals have a higher optimal growth temperature than do large individuals (Ohlberger, 2013; Pörtner & Farrell, 2008). This often leads to increased body growth rates and length‐at‐age of young individuals with warming, while growth rates and length‐at‐age of older individuals may even decrease (Imsland, Sunde, Folkvord, & Stefansson, 1996; Ohlberger, 2013)*.* In addition, size‐specific responses to browning may be caused by changes in resource use over ontogeny (commonly observed in nature: Werner & Gilliam, 1984) coupled with certain prey items and feeding strategies being more negatively affected by brown water color than others (Ask et al., 2009; Jönsson, Ranåker, Nilsson, & Brönmark, 2012). Thus, high temperatures and brown water colors may affect growth of fish differently, depending on the size and feeding stage of the fish.

Water temperature‐ and color‐induced variation in individual body growth will likely influence population‐level attributes such as size‐ and age‐distributions, standing stock biomass, and ultimately fish biomass production (Figure 1). Previously, warming has been shown to lead to smaller mean length (Arranz et al., 2016; Baudron, Needle, Rijnsdorp, & Tara Marshall, 2014; Daufresne, Lengfellner, & Sommer, 2009; Jeppesen et al., 2012) and an increased proportion of small and young individuals in fish populations (Arranz et al., 2016; Daufresne et al., 2009; Jeppesen et al., 2010; Ohlberger, 2013). These changes in size distributions can have large consequences for intraspecific interactions and population dynamics (Brose et al., 2012; Lindmark, Huss, Ohlberger, & Gårdmark, 2018; Ohlberger, Edeline, Vollestad, Stenseth, & Claessen, 2011). Similarly, brown water (or higher DOC) has in some cases been shown to result in a lower mean length‐at‐age (Estlander et al., 2010; Horppila et al., 2010), lower fish population biomass (Finstad et al., 2014) and biomass production (Karlsson et al., 2015, 2009). However, whether these observations from natural systems over relatively small spatial scales (Karlsson et al., 2015, 2009; O'Gorman et al., 2016) or theoretical predictions (Lindmark et al., 2018; Ohlberger et al., 2011) hold for how temperature and water color jointly affect fish biomass production over a large range of natural systems is still unknown.

Here, we studied the combined effects of warmer and browner waters on fish biomass production, using monitoring data from 52 temperate and boreal lakes across large gradients in water temperature and water color. As model species, we used the omnivorous fish species perch (*Perca fluviatilis*), which is a common and often numerically dominant fish species in most of Eurasia (Thorpe, 1977), with economic value (Arlinghaus, Mehner, & Cowx, 2002) and having great impact on lake food webs (Jeppesen et al., 1997; Persson et al., 2003). We hypothesized that fish biomass production is negatively related to both temperature and water color, caused by a reduction in fish biomass and a shift in size distributions toward smaller fish at high temperatures and reduced growth rates in browner waters. To test this hypothesis, we studied both individual‐ and population‐level responses, and how these determine overall fish biomass production (Figure 1). Our study shows that it is necessary to study joint individual‐ and population‐level responses to concurrent warming and browning to understand and predict shifts in fish biomass production in a changing climate.

## 2 MATERIALS AND METHODS

### 2.1 Lake selection

The 52 temperate and boreal lakes studied were obtained from the Swedish National Register of Survey test‐fishing (NORS; National Register of Survey test‐fishing ‐ NORS, 2016). We selected lakes that had Eurasian perch (*P. fluviatilis*; hereafter notated as perch) present. Perch was chosen as a focal species because it is one of the most common fish species in Europe, it often dominates the fish community (numerically and in biomass), and it is present over a wide range of environmental conditions (Craig, 1987; Lehtonen, Rask, Pakkasmaa, & Hesthagen, 2008; Tammi et al., 2003). Like many fish species, perch undergo ontogenetic shifts in diet and habitat use. Larval perch feed exclusively on zooplankton, as juveniles they also feed on benthic macroinvertebrates and at larger sizes also on fish (Amundsen et al., 2003; Hjelm, Persson, & Christensen, 2000; Mittelbach & Persson, 1998). Next to a presence of perch, a further requirement for lakes to be included in the study was a minimum of two years of fish data collected in July or August during 2006 to 2015. July and August represent the main growth season for perch within the particular geographical region (Le Cren, 1958). The chosen lakes also had water temperature and biochemical data sampled in July and/or August for multiple sampling years during the same time period (Miljödata MVM database, https://miljodata.slu.se/mvm/Default.aspx, on 05–12–2016). Lakes larger than 5 km^2^ were excluded to limit variation in lake size. These selection criteria gave us a dataset of 52 small to intermediate sized lakes (area: 0.09–4.89 km^2^) distributed all over Sweden (see supplement Figure S1 and Table S1). Each of the lakes had between 1 and 9 fish species present, and a total of 23 fish species were found in the lakes (Table S2). Perch made up between 22% and 100% of the total fish biomass in each of the selected lakes.

### 2.2 Temperature and water color data

We obtained water geochemical data (Supporting information Table S1) from the Miljödata MVM database (https://miljodata.slu.se/mvm/Default.aspx). The explanatory variables of interest in this study were water temperature and water color. Water temperature and water samples were taken at 0.5 meter depth during July and August between 2006 and 2015 (one sampling occasion per month for a minimum of five years per lake). Mean temperature measured in the lakes during July and August was 19.3°C (15.4–21.4°C, Supporting information Table S1). Water color (brownness) was measured as absorbance of water at 420 nm (Kirk, 1994), where high absorbance is a proxy for brown water. Absorbance was measured using filtered (0.45‐μm filter) water in a 5 cm cuvette, and converted to the Napierian absorption coefficient (a_420_) as recommended by Hu, Muller‐Karger, and Zepp (2002) (see methods in Supplements; hereafter, we use “absorbance” to refer to the Napierian absorption coefficient). Mean absorbance measured across lakes during July and August was 7.0 m^−1^ (0.8–22.2 m^−1^, Supporting information Table S1).

### 2.3 Fish sampling and analyses

#### 2.3.1 Fish sampling

The fish data used from the NORS database were sampled using multimesh gillnets in the benthic and pelagic zones according to a standardized test‐fishing method (Appelberg et al., 1995). In the benthic zone, bottom standing multimesh gillnets were used (net area 45 m^2^) while in the pelagic zone, floating multimesh gillnets were used (net area 82.5 m^2^). The number of gillnets in the benthic zones was standardized according to the size and maximum depth of each lake, and the pelagic zones to maximum depth of each lake, to obtain representative samples of the fish community (CEN, 2015; 35 lakes were deep and large enough to have pelagic gillnets, Table S1). Benthic gillnets used were randomly distributed over the whole lake within fixed depth strata (0–2.9, 3–5.9, 6–11.9, 12–19.9, 20–34.9, 35–49.5, 50–74.9, and >75 m) according to a standardized scheme (CEN, 2015). The total number of benthic nets in a lake ranged between 4 and 48, and the number of pelagic nets ranged between 0 and 20. Gillnet sampling lasted from 19:00 to 07:00 hr, including the periods of dusk and dawn. The method used gives a representative sample of numbers and sizes of most species including perch, except for young‐of‐the‐year individuals (Appelberg et al., 1995; Kurkilahti & Rask, 1996). Most of the fish were caught with benthic nets (60%–100% of the biomass per lake).

All captured fish were identified to species, their total individual length was measured to the nearest mm, and total biomass to the nearest gram of each species per net and mesh size was recorded. In order to obtain individual age‐ and body growth estimates of perch, random subsamples were collected in proportion to the size distribution of the total catch. Perch that were subsampled for aging were measured to the nearest millimeter and weighed to the nearest gram. Otoliths were used for age determination, and operculum bones were used for back‐calculation of annual body growth (Le Cren, 1947; Linløkken, Kleiven, & Matzow, 1991). Not all data were available for all lakes; therefore, the number of lakes used differs for different analyses depending on the response variable studied (N 46‐52), and we report the corresponding N for each analysis in the results.

#### 2.3.2 Fish biomass production

Fish biomass production was represented by biomass production of perch in each lake, measured as gram biomass production per year and net area (g m^−2^ year^−1^), and was derived from the estimated size‐specific annual weight increments of the fish caught in each lake. First, we fitted lake‐specific length‐at‐age and weight‐at‐length relationships for the subset of fish for which we had length (*L*), age (*A*) and weight‐at‐catch (*W*) data, using

(1) $$L=a*A^{b}$$

and

(2) $$W\;\text{=}\;c\;\text{*}\;L^{d}\text{,}$$

where *a*, *b*, *c* and *d* are lake‐specific coefficients (Supporting information Table S3). With these relationships, we calculated the predicted weight and age of each fish at length in the total catch, and subsequently fitted a lake‐specific weight‐at‐age relationship, using

(3) $$W\;\text{=}\;f\;\text{*}\;A^{g}\text{,}$$

where *f* and *g* are lake‐specific coefficients (Supporting information Table S3). With this relationship, we predicted the weight of each individual fish one year prior to the catch year. For fish that were born in the same year as they were caught (i.e., having a predicted age between 0 and 1), the previous weight was set as 0. Then, we calculated the biomass increase of each fish from the previous year until the catch year. The sum of biomass increases across all individuals per lake was divided by the lake‐specific net area, rendering lake‐specific mean fish biomass production (i.e., mean increase in perch biomass) per net area per year (g m^−2^ year^−1^), ensuring that the values of fish biomass production are comparable between lakes.

#### 2.3.3 Population biomass, population abundance, length‐ and age‐distribution

We calculated catch per unit effort (CPUE_biomass_) as an indicator of perch population biomass for each lake by dividing the total biomass caught over multiple years, by the total net area and fishing time (g m^−2^ night^−1^) used in those years. Population abundance (CPUE_N_) was calculated by dividing the total number of individuals caught over multiple years, by the total net area and fishing time (N m^−2^ night^−1^) used in those years. For each lake, we calculated mean total length (mm) and skewness of total length of the perch in catches across all years (2006–2015). Skewness in length was used as a measure of the relative proportion of small to large individuals, a higher skewness signifying a greater proportion of small individuals in the population. We calculated mean and skewness in age of each population based on all perch in the catch in each lake (see *Fish biomass production* for age estimates). Skewness in age is a measure of the relative proportion of young to old individuals, a higher skewness corresponding to a greater proportion of young individuals.

#### 2.3.4 Length‐at‐age and length‐specific growth rates

From the subsamples of perch from each lake where age data were available, we calculated mean length‐at‐age (mm) at catch of age 1 and 6. Length‐at‐age 1 represents growth during the first year and length‐at‐age 6 is a result of the growth during the first five years of life. Length‐specific growth rates of perch were estimated using back calculated length‐at‐age data from operculum bones (Holmgren & Appelberg, 2001). Growth during the catch year was excluded since this was not a full year of growth. Per lake we calculated individual length‐specific growth rates for all ages of fish using

(4) $$G_{L,t-1}=\;\left(\left(L_{t}\;-\;L_{t-1}\right)/\;L_{t-1}\right),$$

where *G_L,t_*_−1_ is the annual length‐specific growth at the length (*L*) the fish had at age *t *− 1. Using these individual annual length‐specific growth values, we subsequently estimated a general length‐specific growth rate function for each lake (*G_L_*_,lake_), by fitting the individual annual length‐specific growth values from equation (4) as an exponential function of body length (in the previous year) across all individuals and catch years in that lake using

(5) $$G_{{L,t-1,}_{\mathrm{lake}}}=\alpha_{\mathrm{lake}}\;*\;{\text{e}}^{\beta_{\mathrm{lake}}\;*\;L_{t-1}},$$

with a lake‐specific scalar (*α*_lake_) and exponent (*β*_lake_; Supporting information Table S3). Applying equation (5), we calculated length‐specific growth rates for specific lengths and lakes, here for 170 mm perch, representing adult individuals.

### 2.4 Abiotic and biotic data for robustness tests

In addition to water temperature and absorbance, two abiotic covariates representing factors shown to influence fish abundance and community structure in lakes (Karlsson et al., 2009; Mehner, Diekmann, Bramick, & Lemcke, 2005; Persson, Diehl, Johansson, Andersson, & Hamrin, 1991), and that were available for all lakes, were selected a priori; mean depth (D, proxy for lake morphology) and total phosphorus (P, proxy for productivity). The use of only these two specific abiotic covariates to represent lake morphometry and productivity was done to avoid covariation among explanatory variables, for example, mean depth was correlated with lake area (*R* = 0.295, *p* < 0.05) and total phosphorus was strongly correlated with total nitrogen (*R* = 0.757, *p* < 0.0001). Also chlorophyll *a* concentration, which has been measured in all study lakes, was strongly correlated with total phosphorous (*R* = 0.895, *p* < 0.0001). Chlorophyll *a* is, however, in this case only a measurement of suspended phytoplankton in the pelagic area (Swedish Environmental Protection Agency ‐ SEPA, 2007), and not of benthic algae which are likely more affected by water color (Seekell, Lapierre, Ask et al., 2015; Vasconcelos et al., 2016). Thus, we chose to include only mean depth and total phosphorus as abiotic covariates in our robustness tests. The above data were also obtained from the Miljödata MVM database (https://miljodata.slu.se/mvm/Default.aspx). Water samples were taken at 0.5 meter depth during July and August between 2006 and 2015 (one sampling occasion per month for a minimum of two years per lake). Mean depth across lakes was 5.6 m (range 1.4–14.1 m, Supporting information Table S1) and mean total phosphorus across lakes during July and August was 10.4 µg/L (range 2.7–30.7 µg/L, Table S1). All water samples were collected and analyzed according to standard limnological methods (https://www.slu.se/en/departments/aquatic-sciences-assessment/laboratories/vattenlabb2/, on 07–05–2018).

In addition to abiotic factors, presence and abundance of other fish species may influence perch biomass production (Eloranta et al., 2016), for example, through trophic interactions. Here, we choose to focus on the influence of roach *(Rutilus rutilus)* population biomass (as a covariate) because (a) roach and perch interact strongly, both through predator–prey and competitive interactions (Persson, 1987; Persson & Eklöv, 1995), (b) roach was the most commonly occurring competitor and prey fish species in our lake data set, found in 42 of the lakes and (c) had a variable but relatively high population biomass in most of the lakes (0.25%–55% of the fish biomass per lake; Supporting information Table S2). Roach population biomass was calculated in the same way as for perch. With a linear regression model, we tested if roach biomass was driven by temperature and absorbance (only including lakes where roach is present), and did not find a significant relationship (*p*_temp_ = 0.181, *p*_abs_ = 0.240), making it possible to include it as a covariate in the robustness analyses.

### 2.5 Statistical analyses

#### 2.5.1 Hypothesis tests

We used multiple linear regression analyses to test the hypotheses that water temperature and absorbance affect fish biomass production, population biomass, population abundance, mean population length, skewness in length, mean population age, skewness in age, length‐at‐age 1 and 6, and length‐specific growth at 170 mm, (lm[response variable ~ water temperature × absorbance]). Prior to analyses, we used variance inflation factor analysis (VIF, vif function from the *car* package, Fox & Weisberg, 2011), to make sure the level of multicollinearity between explanatory variables and covariates was low (VIF <4). Normality of the residuals was tested with a Shapiro–Wilk test and by visual inspection. All response variables were ln‐transformed (except for skewness of length and age) to normalize the data. Outliers (determined by Cook's distances, cookd function from the car package (Fox & Weisberg, 2011)) were removed prior to analysis. In only one case were outliers identified (skewness in age), and their exclusion had no influence on the results. Selection of explanatory variables was based on significance levels *p* < 0.05 (two‐sided tests). Nonsignificant interaction terms were removed before testing for an effect of temperature and absorbance. We performed a Moran I analysis with the *ape* package (Paradis, Claude, & Strimmer, 2004) to test for spatial autocorrelation in the residuals, but found no significant residual spatial autocorrelation in any of the models (results not shown). All statistical tests were done in R 3.4.2 (R Core Team, 2017).

#### 2.5.2 Robustness analyses

As a test of the robustness of the linear regression analyses, we performed model selection on four different linear regression models for each response variable with either temperature, absorbance, temperature + absorbance, or temperature × absorbance, and with or without two different sets of abiotic and biotic covariates (mean depth + total phosphorus or roach population biomass). If the best model(s) following model selection (ΔAICc <2) included the same significant explanatory variables (temperature and/or absorbance) identified using linear regression analyses (that did not include covariates, Table 1), and those variables were the same irrespective of whether either set of (abiotic or biotic) covariates were included or not, this was recognized as a sign that the influence of the explanatory variables was robust to the choice of model structure (i.e., including or excluding variables representing lake morphology, lake productivity, and trophic interactions with other species).

**Table 1:** Multiple linear regression analyses on the effects of temperature (°C) and absorbance (water color, *a*_420_, m^−1^) on response variables (lm[response variable ~ water temperature + absorbance]). N indicates the number of lakes included (differ because of data availability, see methods) and LN that the response variable was ln‐transformed prior to analysis. D is the direction of change, zeros indicating nonsignificant relationships. A positive skewness in length/age represents a higher proportion of small/young individuals. (*p < 0.05, **p < 0.01, ***p < 0.001, nonsignificant results in bold). In no case was there a significant interaction between temperature and absorbance

Response variable	Temperature		Absorbance		*R* ^2^
	D	F	D	F	
Fish biomass production (LN) *N* = 46	**−**	*F*_(1,43)_ = 5.27*	**−**	*F*_(1,43)_ = 10.59**	**0.29**
Population biomass (LN) *N* = 52	**−**	*F*_(1,49)_ = 6.01*	0	*F*_(1,49)_ = 2.96	**0.16**
Population abundance (LN) *N* =52	0	*F*_(1,49)_ = 2.18	0	*F*_(1,49)_ = 0.99	**0.06**
Mean length (LN) *N* = 49	**−**	*F*_(1,46)_ = 8.51**	0	*F*_(1,46)_ = 1.41	**0.19**
Skewness (positive) in length *N* = 49	**+**	*F*_(1,46)_ = 16.76***	0	*F*_(1,46)_ = 0.08	**0.27**
Mean predicted age (LN) *N* = 46	**−**	*F*_(1,43)_ = 14.62***	**+**	*F*_(1,43)_ = 4.23*	**0.29**
Skewness (positive) in age *N* = 42	**+**	*F*_(1,39)_ = 15.91***	0	*F*_(1,39)_ = 0.0084	**0.29**
Length‐at‐age 1 (LN) *N* = 49	**+**	*F*_(1,45)_ = 4.83*	**−**	*F*_(1,45)_ = 7.48**	**0.20**
Length‐at‐age 6 (LN) *N* = 49	0	*F*_(1,45)_ = 0.05	**−**	*F*_(1,45)_ = 28.29***	**0.39**
Length‐specific growth rate at 170 mm body length (LN) *N* = 49	0	*F*_(1,46)_ = 0.61	**−**	*F*_(1,46)_ = 6.86*	**0.15**

## 3 RESULTS

### 3.1 Fish biomass production

**Figure 2:**
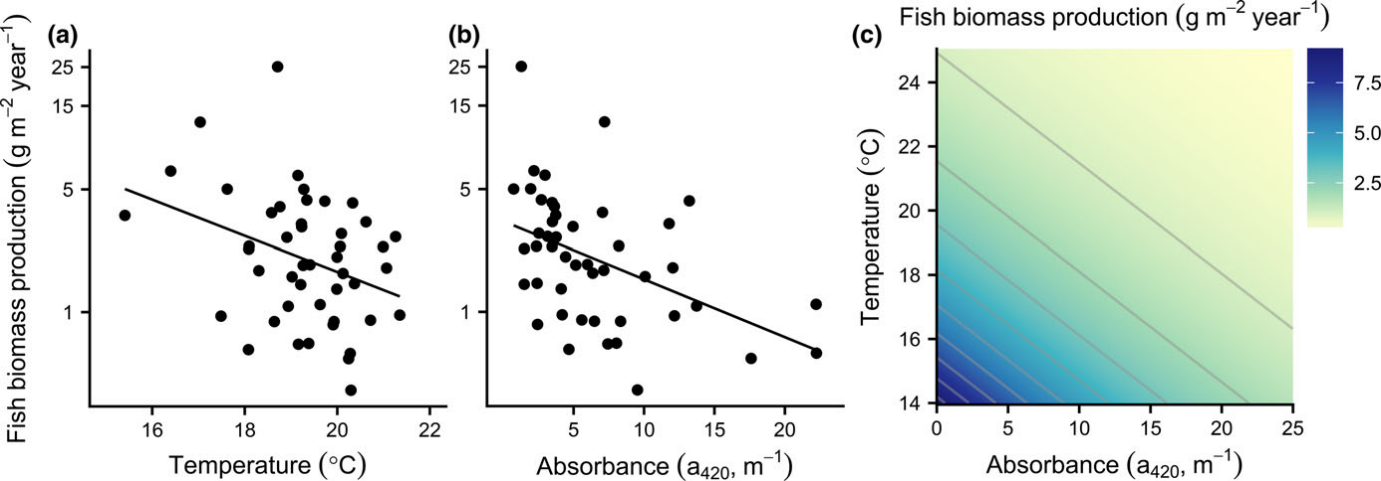
Fish biomass production. Relationships between fish biomass production (g m^−2^ year^−1^) and (a) temperature and (b) absorbance. Black dots represent individual lakes and solid black regression lines significant linear relationships (see Table 1). In (c) predicted fish biomass production is shown as a function of temperature and absorbance based on the best linear regression model: ln(fish biomass production) ~ temperature + absorbance, see Table 1) [Colour figure can be viewed at http://wileyonlinelibrary.com]

Perch biomass production was negatively related to both higher water temperature and absorbance (Table 1, Figure 2), with the lowest values observed in warm and brown lakes (Figure 2c). This pattern holds also when we exclude fish caught in the pelagic area which were not sampled in all lakes (*F*_T(1,43)_ = 7.82**, *F* *a*_420_(1,43) = 17.53***, *R* ^2^ = 0.40). A lake with the mean water temperature and absorbance (*T* = 19.3°C, *a*_420_ = 7.0 m^−1^) is expected to have three times higher fish biomass production (1.93 compared to 0.64 g m^−2^ year^−1^) than a relatively warm and brown lake (*T* = 22.3°C, *a*_420 _= 14.0 m^−1^), while fish biomass production is predicted to only be halved (1.04 g m^−2^ year^−1^) with the same change in temperature without a simultaneous change in water color, and to decrease with a third (to 1.18 g m^−2^ year^−1^) with a corresponding change in absorbance only.

### 3.2 Population biomass, population abundance, length‐ and age‐distribution

**Figure 3:**
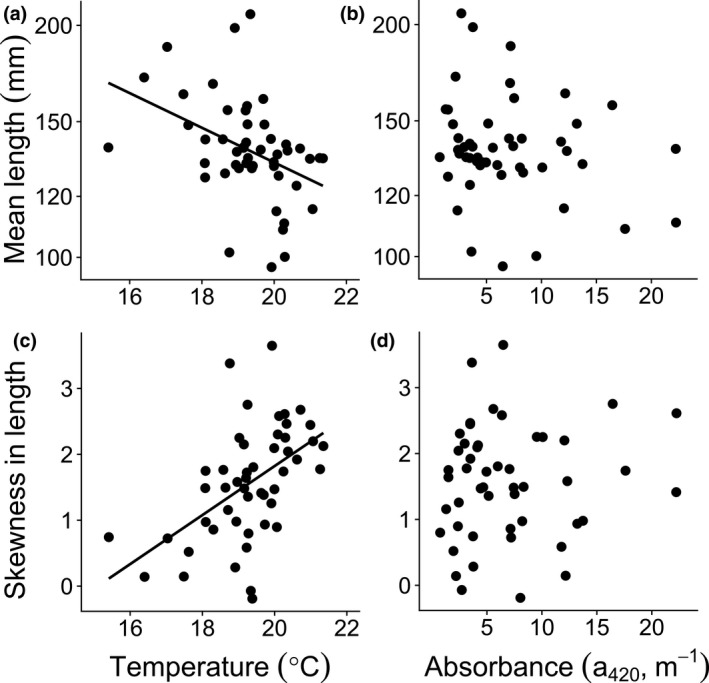
Length distributions. Relationships between mean length (in mm, top graphs) and (a) temperature and (b) absorbance and between skewness in length (bottom graphs) and (c) temperature and (d) absorbance. Black dots represent individual lakes and solid black regression lines significant linear relationships. See Table 1 for statistical analyses

**Figure 4:**
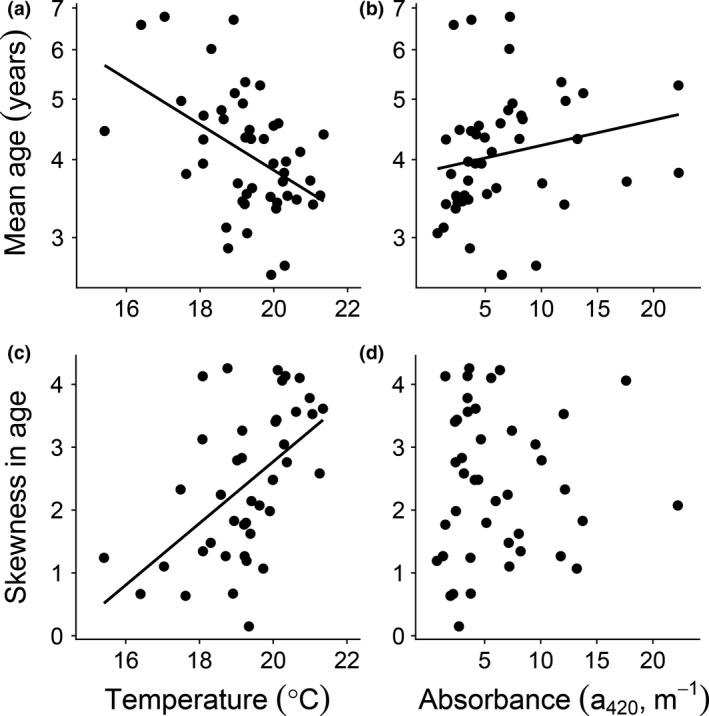
Age distributions. The relationship between predicted mean age (in years, top graphs) and (a) temperature and (b) absorbance and the relationship between skewness in age (bottom graphs) and (c) temperature and (d) absorbance. Black dots represent individual lakes and solid black regression lines significant linear relationships. See Table 1 for statistical analyses

Population biomass was lower at high than at low water temperatures but was not affected by absorbance (Table 1, Supporting information Figure S2a). In contrast, population abundance was not affected by either high temperatures or brown water. Mean body length and skewness in length in the fish populations were both negatively affected by higher water temperatures (Table 1, Figure 3a), but did not respond to higher absorbance, suggesting a higher proportion of small individuals in high temperature lakes irrespective of water color (Table 1, Figure 3c). Both higher water temperature and absorbance affected mean age (Table 1, Figure 4a, b), but in different directions; water temperature had a negative, while absorbance had a positive effect. Higher water temperature also had a negative effect on skewness in age, suggesting that there was a higher proportion of young individuals in warm lakes (Table 1, Figure 4c).

### 3.3 Individual body growth and length‐at‐age

**Figure 5:**
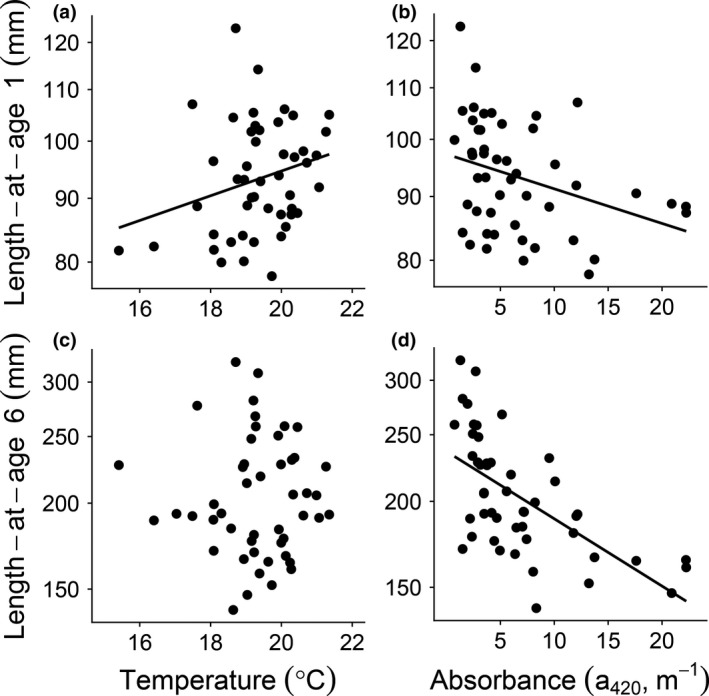
Length‐at‐age 1 and 6. The relationships between length‐at‐age 1 (in mm, top graphs) and (a) temperature and (b) absorbance and between length‐at‐age 6 (in mm, bottom graphs) and (c) temperature and (d) absorbance. Black dots represent individual lakes and solid black regression lines significant linear relationships. See Table 1 for statistical analyses

Individual body growth and responses in length‐at‐age to temperature and absorbance depended on length and age (Table 1). Higher water temperature positively affected the mean length of 1‐year‐old fish (equivalent to first year body growth), while higher absorbance instead had a negative effect on first year body growth rates (Table 1, Figure 5a, b). In contrast, while also the mean length of 6‐year‐old fish was negatively affected by higher absorbance (Table 1, Figure 5d), temperature had no effect. Similarly, high absorbance negatively affected length‐specific growth of large (170 mm), adult perch (Table 1, Supporting information Figure S3b) whereas high temperature had no effect.

### 3.4 Robustness analyses

For all response variables, model selection resulted in that at least one of the selected best models (ΔAICc ± 2) included the explanatory variables (temperature and/or absorbance) identified to be significant in the multiple linear regression analysis, irrespective of covariates (mean depth and total phosphorus or roach population biomass) being included or not (compare Table 1 and Supporting information Tables S4, S5). Also, adding these covariates, representing lake morphology, productivity, and trophic interactions, only marginally improved model fits (Supporting information Tables S4 and S5, except for population biomass and abundance). Thus, our results concerning the influence of temperature and absorbance on response variables are robust to the choice of model structure.

## 4 DISCUSSION

**Figure 6:**
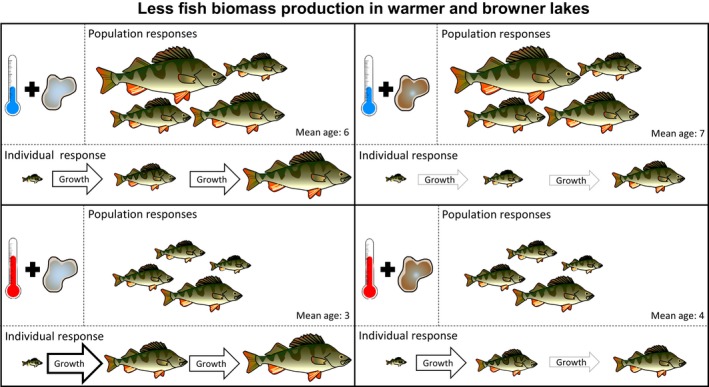
Fish biomass production in warmer and darker lakes. As the water gets warmer and browner, fish biomass production decreases through different pathways. With warming alone (from top to bottom left), population biomass decreases, and there is a shift toward smaller and younger individuals. Young but not old individuals exhibit higher growth rates. As lakes get browner (but not warmer; from top left to top right), mean age increases slightly, but there is no shift in population biomass or mean length. Body growth of both small and large individuals is reduced. As lakes get both warmer and browner (from top left to bottom right), there is a strong decrease in fish biomass production due to reduced population biomass, a shift toward smaller and younger individuals and slower body growth

Our results show that both high water temperature and high absorbance (brown water color) during summer negatively affect fish biomass production across a large number of lakes. Considering that temperate and boreal lakes are predicted to become both warmer and browner because of climate change (Larsen et al., 2011; Roulet & Moore, 2006; Weyhenmeyer et al., 2016), our results suggest a potential drop in future lake fish biomass production in large parts of Europe, especially given that perch is the dominant fish species in many European lakes (Craig, 1987; Lehtonen et al., 2008; Tammi et al., 2003). Accordingly, only assessing impacts of temperature, ignoring simultaneous changes in water color (or vice versa), would underestimate the impact of climate change on fish populations and production. In addition, we show how the negative effects of high temperature and brown water color on fish biomass production emerge through different routes, that is, the temperature effect goes mainly via population‐level responses and the brown water effect mainly through individual‐level responses (Figures 1 and 6). Overall, our study shows that in order to predict climate change effects on fish biomass production or its variation across lake environments, we simultaneously need to consider multiple climate stressors, as well as both individual‐ and population‐level responses.

Our results show that the lower fish biomass production in warm compared to cold lakes most likely results from smaller population biomass, a lower mean length and age, and a higher proportion of small and young individuals (Figure 6). In contrast, the lower fish biomass production in brown compared to clear lakes is mainly explained by reduced body growth and length‐at‐age (Figure 6). The link from low population biomass to low fish biomass production in warm lakes is simply explained by less biomass available for growth. The lower fish biomass in warm lakes is, in turn, most probably a consequence of the observed shift toward a higher proportion of small‐bodied individuals, which is in line with findings of previous studies (Daufresne et al., 2009; Jeppesen et al., 2010). The shift to smaller/younger fish is likely related to higher mortality of larger and older individuals (Sandström, Neuman, & Thoresson, 1995) and/or earlier maturation at a smaller size at high temperatures (Atkinson, 1994; Sandström et al., 1995), which reduces size at old ages due to slower body growth after maturation (Charnov, Turner, & Winemiller, 2001). The observed shift toward smaller fish in warm lakes likely leads to a lower population fecundity, given that small individuals have a lower reproductive output than larger and older individuals (especially females; Berkeley, Chapman, & Sogard, 2004; Dubois, Gillet, Bonnet, & Chevalier‐Weber, 1996). While leading to lower fish biomass production, browner water color did not have a significant negative effect on population biomass. This contradicts some previous studies that found negative relationships between light condition (Karlsson et al., 2009) or DOC (Karlsson et al., 2015) and fish population biomass. On the other hand, Finstad et al. (2014) found a positive effect on fish population biomass as lake DOC increased from very low levels (i.e., a hump‐shaped relationship), likely because of a positive effect of subsidies of organic carbon and nutrients associated with DOC (See also Kelly et al., 2016; Seekell, Lapierre, & Karlsson, 2015; Zwart et al., 2016). However, direct comparisons to these particular studies are difficult, as we used either a different measurement of water color (absorbance), a much wider geographical range of lakes of which some had higher fish diversity than in previous studies, and because we (only) used the dominant fish species, perch, as our focal fish species. Still, even though we found no effect of brown water color on population biomasses, we found strong individual‐level responses, with perch in brown lakes having slower body growth and smaller length‐at‐age than perch in clear lakes. The latter explains the lower fish biomass production in brown lakes. The negative relationship between water color and body growth across the large geographical gradient in our study supports and generalizes the findings of previous smaller‐scale lake studies (Estlander et al., 2010; Horppila et al., 2010). This relationship is possibly due to decreased resource production (Ask et al., 2009; Karlsson et al., 2009; Seekell, Lapierre, Ask et al., 2015; F. Rivera Vasconcelos et al., 2018) and visibility (Jönsson et al., 2011; Ranåker et al., 2012) in brown lakes. Overall, we found additive negative effects of water temperature and color on fish biomass production, leading to many times lower fish biomass production in warm and brown lakes compared to cold and clear lakes.

Not only did water temperature and color differ in their effect on individual performance but responses also varied over ontogeny. Body growth of young/small but not old/large fish was positively affected by high temperatures. The observed shift in body growth responses to temperature over ontogeny can be explained by that biological rates like metabolism and feeding generally increase with both temperature and body size (Brown et al., 2004; Rall et al., 2012), but often not at the same rate (Lindmark et al., 2018). This generally results in small fish, including perch (Ohlberger et al., 2011), having a higher temperature optimum for growth than large individuals (Imsland et al., 1996; Ohlberger, 2013). Thus, in such a scenario, small but not large individuals will exhibit increased growth rates at high temperatures (Daufresne et al., 2009; Ohlberger, 2013), similar to what we found in our study. Even though small individuals exhibit higher growth rates and are energetically more efficient (i.e., higher rate of fish biomass production per unit biomass) than large ones (Byström & García‐Berthou, 1999; Persson & De Roos, 2013), the absolute growth of a small individuals is lower. Given that the shift to smaller individuals did not increase the total number of individuals, the combination of smaller individuals and lower overall population biomass led to a lower fish biomass production in warm lakes (Figure 6).

In contrast to observed body growth responses to high temperatures, brown water color had a negative effect on individual body growth and length irrespective of age, but more so on old/large individuals. The length‐specific responses to variation in water color could be a consequence of smaller perch mainly feeding on zooplankton, while older and larger individuals feed more on benthic and fish prey (Hjelm et al., 2000; Mittelbach & Persson, 1998). The production of benthic invertebrates is known to decrease with browning (Estlander et al., 2010; Karlsson et al., 2009) due to lowered benthic primary production, while pelagic production is less affected (Ask et al., 2009), or even positively affected by a higher nutrient concentrations in brown waters (Vasconcelos et al., 2016). Thus, larger fish individuals may be more limited by a reduction in prey availability than small individuals in brown waters. Moreover, the higher prey density needed for large fish to sustain themselves (Byström & García‐Berthou, 1999) may contribute to why browner water, given an expected decrease in prey availability, has more severe negative effects on large individuals. Also, the fact that feeding on fast moving fish prey (i.e., food for large perch) is more negatively affected by reduced visibility than feeding on slow moving zooplankton (i.e., food for small perch)(Jönsson et al., 2012) may contribute to the stronger negative effect of brown waters on large fish. Slower growth, especially of large individuals, in turn, reduces fish biomass production (Figure 6). As lakes are projected to get both warmer and browner (Dokulil, 2014; Weyhenmeyer et al., 2016), the small positive effect of increased growth rates of small individuals on fish biomass production in warm waters will likely be overridden by decreased growth rates of both small and large individuals in browner waters, resulting in an overall decrease in fish biomass production (Figure 6).

Using a space‐for‐time approach, our results indicate that fish biomass production will likely decrease with warming and browning of lakes, which is expected under climate change (Dokulil, 2014; Larsen et al., 2011; Roulet & Moore, 2006; Weyhenmeyer et al., 2016; Whitehead et al., 2009). Global surface temperatures are predicted to increase between 0.3 and 4.8°C until 2,100, with a higher than average increase in boreal and temperate regions (IPCC, 2014). Water color (absorbance, *a*_420_) is predicted to increase by a factor of 1.1 to 7.6 already until 2030, depending on for example, lake retention time (Weyhenmeyer et al., 2016). Thus, also with relatively conservative estimates, as exemplified in the results (*T* = 19.3 to 22.3°C, *a*_420_ = 7.0–14.0), fish biomass production may decrease by as much as a factor 3. Consequently, while warming may have significant effects on biomass production in the long term, browning may change fish biomass production also over relatively short time scales. Also, whereas temperature change cannot be managed on the local scale, the link between browning and land use (Larsen et al., 2011; Sobek, Tranvik, Prairie, Kortelainen, & Cole, 2007; Weyhenmeyer et al., 2016) suggests that management actions on the local or regional scale may mitigate some of the negative effects of browning in lakes. Although the assumption that spatial relationships can correctly project temporal ones may not always be warranted, it is the best at hand as long time series (>30 years) covering multiple lakes exposed to gradual change in temperature and/or brown water color to different degrees are generally lacking.

Fish biomass production responses to water temperature and color are likely to be species‐specific, for example, due to different temperature optima (Pörtner & Farrell, 2008), and here, we only looked at a single, representative species (perch). However, we still expect the negative effects on fish biomass production to be generally valid for systems dominated by visually hunting fish, fish relying on a high production of benthic prey, and those close to their temperature optima. Also, as perch is a common and numerically dominant fish species in many Eurasian lakes (Craig, 1987; Lehtonen et al., 2008; Tammi et al., 2003), a decrease in perch biomass production will in many lakes impact overall fish production.

Although our results indicate that high temperatures and brown waters lead to lower fish biomass production, fish communities are simultaneously affected by other ongoing stressors linked to climate change (e.g., eutrophication and deoxygenation; Whitehead et al., 2009), which may also contribute to some of the unexplained variation in our study. Still, despite not taking these other abiotic variables into account, we find significant negative effects of warm temperatures and brown water color on fish biomass production across a large number of lakes. Furthermore, we showed that the negative effect of high temperatures and brown waters was present over a wide range of competitive environments. Roach, a key competitor of perch (Persson, 1987; Persson & Eklöv, 1995), ranged from not being present at all to being more abundant than perch in our study lakes. Still, inclusion of this large variation in competitive environment (Supporting information Table S5) did not change the effects of temperature and water color on fish biomass production or underlying variables (Figure 6). Unfortunately, for the lakes used in this study, we lacked appropriate data on primary production and invertebrate prey availability making it difficult to draw conclusions about the extent to which shifts in lower trophic level production contributed to shifts in perch biomass production.

Our findings suggest that the projected warming and browning of temperate and boreal lakes can have negative consequences for fish biomass production, with potential implications for ecosystem function, fisheries, and food security. This is to our knowledge the first study looking at the effect of multiple climate stressors on fish biomass production across a large number of lakes, showing that fish biomass production is likely to be much more negatively affected than predicted by studies taking only single climate stressors into account. We also conclude that water temperature and water color affect fish biomass production through different pathways, via differential effects on population biomass, length‐ and age‐distribution, and body growth of individuals, with impacts also varying over ontogeny. In conclusion, this study shows that in order to predict future climate change impacts on fish biomass production and adapt adequate management strategies, we simultaneously need to consider the effects of multiple climate stressors on both individuals, populations, and communities.
