# Supplementary material for: Warmer and browner waters decrease fish biomass production
Source: Glob Chang Biol. 2019 Feb 10;25(4):1395–408. doi: 10.1111/gcb.14551 (PMC6850179; doi:10.1111/gcb.14551)
Supplement: Supplementary file 1 [file GCB-25-1395-s001.pdf]

Supporting information for the study:

Warmer and browner waters decrease fish biomass production

Renee M. van Dorst[1], Anna Gårdmark[2], Richard Svanbäck[3], Ulrika Beier[4,5], Gesa A. Weyhenmeyer[6], Magnus Huss[2]

1. Swedish University of Agricultural Sciences, Department of Aquatic Resources, Institute of Coastal Research, Skolgatan 6, SE-742 42 Öregrund, Sweden. Corresponding author: tel: 0046-104784176, renee.van.dorst@slu.se
2. Swedish University of Agricultural Sciences, Department of Aquatic Resources, Skolgatan 6, SE-742 42 Öregrund, Sweden
3. Department of Ecology and Genetics; Animal Ecology, Evolutionary Biology Centre, Science for Life Laboratory, Uppsala University, Norbyvägen 18d, 75236 Uppsala, Sweden
4. Swedish University of Agricultural Sciences, Department of Aquatic Resources, Institute of Freshwater Research, Stångholmsvägen 2, SE-178 93, Drottningholm, Sweden
5. Wageningen Marine Research, P.O. Box 68, 1970 AB IJmuiden, the Netherlands
6. Department of Ecology and Genetics/Limnology, Evolutionary Biology Centre, Uppsala University, Norbyvägen 18d, 75236 Uppsala, Sweden

Supplementary methods

Absorbance, Napierian coefficient

We calculated the Napierian coefficient ($a_{420,}$ in m^{-1}) from the absorbance of filtered lake water (0.45 µm filter) at 420 nm in a 5cm cuvette ($AbsF_{420nm/5cm}$) as:

$$a_{420} = (AbsF_{420nm/5cm} * \ln(10))/\mathrm{OL} \qquad (1)$$

where a_{420} is the Napierian coefficient, $AbsF_{420nm/5cm}$ the measured absorbance of filtered water at 420 nm, and OL is the optical path-length (in m).

Supplementary figures

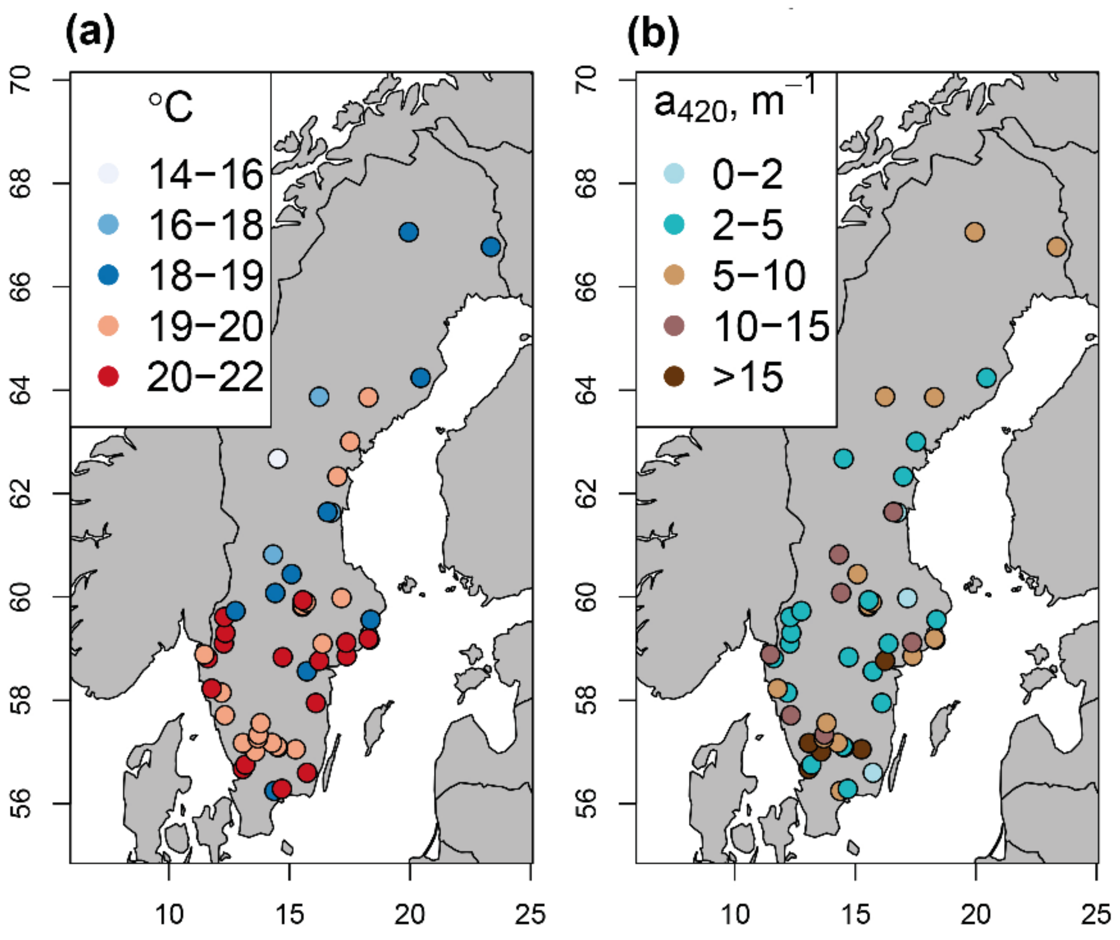

Figure S1 Maps of the 52 Swedish temperate and boreal study lakes. **a** The water temperature and **b** the water colour (absorbance) gradient of the 52 study lakes.

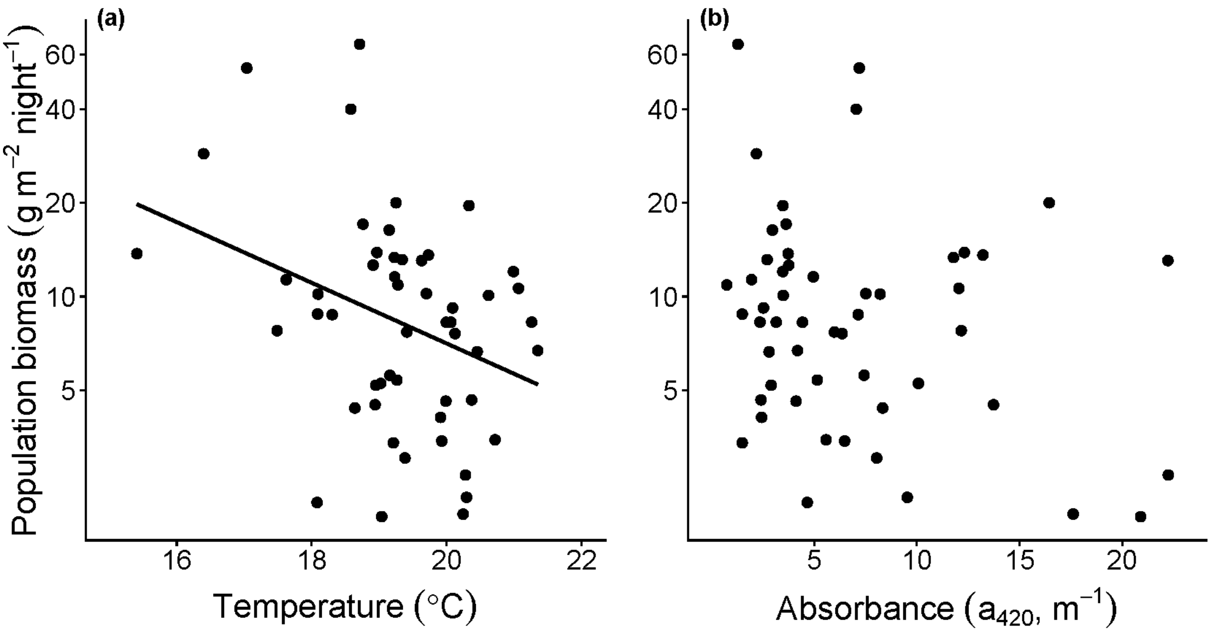

Figure S2 Population biomass. Relationships between perch standing stock biomass and **a** temperature and **b** absorbance. Black dots represent individual lakes and the solid black regression line a significant linear relationship. See table 1 for statistical analyses.

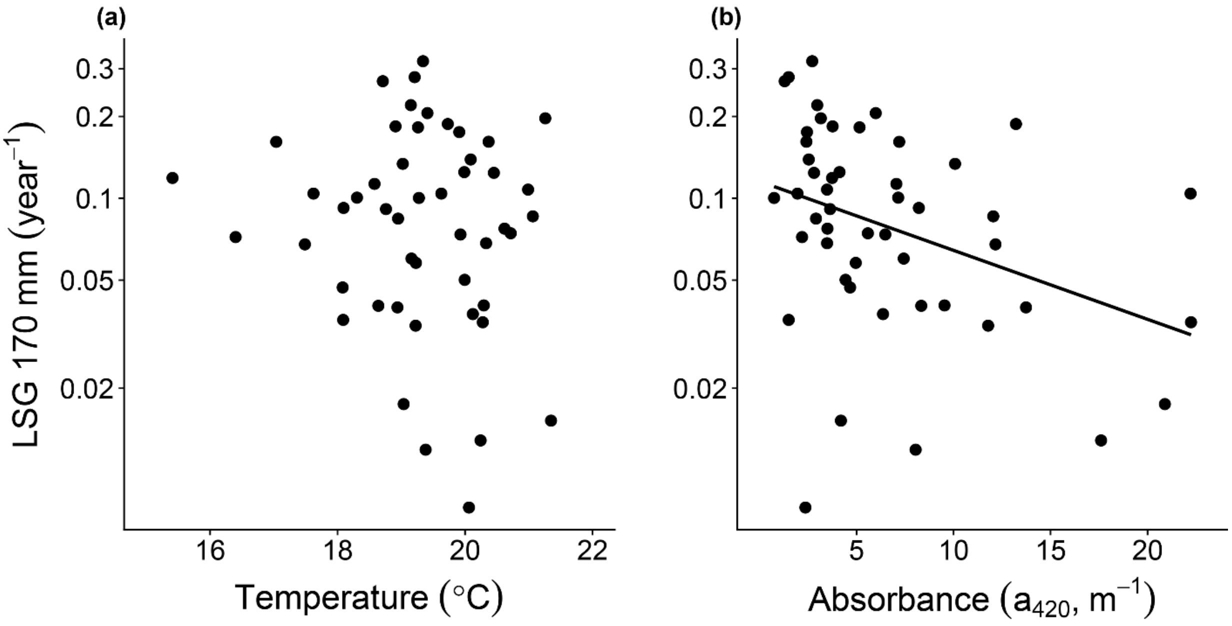

Figure S3 Length-specific growth. Relationships between length-specific body growth (LSG, year^{-1}) of 170 mm perch and **a** temperature and **b** absorbance. Black dots represent individual lakes and the solid black regression line a significant linear relationship. See table 1 for statistical analyses.

Supplementary tables

Table S1 Environmental conditions, physical characteristics and location of the 52 study lakes. Mean values of temperature (°C), absorbance (a_{420}, m^{-1}) and total phosphorus (P, µg/l) are based on samples collected between 2006 and 2015. All lakes had benthic sampling of the fish community, part of them also had pelagic sampling (indicated by 1).

Lake	Latitude (WGS84)	Longitude (WGS84)	Absorbance (a_{420})	Temperature (°C)	P (µg/l)	Mean depth (m)	Area (ha)	Pelagic sampling
Bäen	56.2460	14.3775	8.34	18.64	15.98	3.4	58	0
Örsjön	56.2864	14.6851	4.19	21.35	9.80	3.5	18	0
Brunnsjön	56.5972	15.7281	22.24	20.28	12.39	5.3	10	1
Stora Skärsjön	56.6712	13.0658	2.36	20.06	8.06	3.9	32	1
Gyltigesjön	56.7532	13.1740	20.90	19.04	15.13	9.1	40	1
Harasjön	57.0074	13.5792	22.22	19.63	30.74	2.3	61	0
Hjärtsjön	57.0515	15.2573	1.29	18.71	3.80	3.4	137	0
Fiolen	57.0920	14.5296	2.97	19.15	13.45	3.9	156	1
Gyslättasjön	57.1080	14.4835	8.04	19.38	14.45	2.8	32	1
Älgarydssjön	57.1757	14.2727	16.44	19.25	26.10	1.4	34	0
Nässjön	57.1723	13.0674	6.48	19.93	16.00	2.7	52	0
Hagsjön	57.2644	13.6865	13.22	19.73	8.50	4.6	24	0
Hagasjön	57.3372	13.7114	5.99	19.41	8.54	3.7	11	0
Stengårdshulta-sjön	57.5578	13.8020	10.08	19.02	7.29	7.1	489	1
Stora Härsjön	57.7095	12.3217	2.41	20.37	4.14	14.1	257	1
Allgjutten	57.9479	16.0963	2.44	19.91	3.89	11.7	18	1
Fräcksjön	58.1482	12.1812	5.58	20.72	9.76	4.1	28	1
Granvattnet	58.2260	11.7707	3.63	18.76	24.42	1.6	18	0
Geten	58.5610	15.7217	17.61	20.24	20.88	3.6	20	0
Skärgölen	58.7631	16.2339	2.80	20.45	5.88	7	18	1
Långsjön	58.8346	14.7208	9.53	20.30	11.48	4.2	67	1
Björken	58.8562	17.3702	3.15	21.26	7.62	12.5	137	1
Rotehogstjärnen	58.8150	11.6124	11.78	19.23	13.58	3.6	16	0
Ejgdesjön	58.8854	11.4709	2.71	19.34	3.56	7	86	1
Älgsjön	59.0949	16.3694	12.05	21.06	27.35	2.5	36	0
Stora Envättern	59.1149	17.3535	3.47	20.99	6.89	5.4	38	1
Västra Solsjön	59.1009	12.2763	1.50	19.21	2.71	12.3	184	1
Stensjön	59.1745	18.3244	4.12	19.99	5.74	9.1	39	1
Långsjön	59.1903	18.2969	6.36	20.12	7.75	3.8	9	0
Årsjön	59.1927	18.2781	3.48	20.33	6.60	3.8	21	0
Bysjön	59.3024	12.3399	2.91	18.95	11.92	7.4	113	1
Tärnan	59.5570	18.3660	3.50	20.62	11.01	4.3	105	1
Ulvsjön	59.6098	12.2937	4.67	18.08	7.08	10	49	1
Örvattnet	59.7257	12.7579	1.50	18.09	4.53	9	80	1
Lien	59.8087	15.5288	5.15	19.26	4.00	7.8	149	1
Övre Skärsjön	59.8371	15.5503	7.43	19.16	5.84	6.1	169	1

Dagarn	59.8970	15.6867	2.53	20.09	5.32	5.1	172	1
Västra Skälsjön	59.9347	15.5529	0.74	19.28	3.32	6.6	43	1
Siggeforasjön	59.9779	17.1589	12.31	18.97	15.06	4.2	76	0
Skifsen	60.0755	14.4094	8.21	18.09	11.00	2.6	32	1
Tryssjön	60.4406	15.0880	12.16	17.49	7.26	7.2	30	1
Rädsjön	60.8198	14.3184	1.95	17.62	6.83	8.8	58	1
Källsjön	61.6331	16.7354	13.73	18.94	9.08	7.1	24	1
Stensjön	61.6428	16.5753	4.96	19.23	5.59	4.3	59	1
Väster Rännöbodsjön	62.3301	16.9872	4.43	20.00	12.74	6.2	48	1
Stor-Backsjön	62.6750	14.5135	7.20	17.04	12.21	2.3	206	0
Valasjön	63.0024	17.5153	7.52	19.70	10.05	9	178	1
Degervattnet	63.8728	16.2293	3.76	18.91	5.46	5.1	158	1
Remmarsjön	63.8620	18.2726	7.14	18.31	8.61	5	140	1
Bjännsjön	64.2389	20.4387	7.05	18.58	10.20	1.7	48	0
Pahajärvi	66.7709	23.3529	2.19	16.40	11.87	3.9	132	1
Jutsajaure	67.0590	19.9436	3.73	15.41	7.67	2.3	113	1

Table S2 Fish species found in the study lakes during the sampling period. English name, latin name, the number of lakes in which the species was found, and the mean population biomass (g*m^{-2}*night^{-1}) per lake in the lakes where the species occurred.

**Fish species**	**Latin name**	**Number of lakes present**	**Mean population biomass (g*m^{-2}*night^{-1})**
Eurasian Perch	*Perca fluviatilis*	52	11.48
Northern pike	*Esox lucius*	47	2.36
Roach	*Rutilus rutilus*	42	5.63
Ruffe	*Gymnocephalus cernua*	23	0.23
Common rudd	*Scardinius erythrophthalmus*	13	0.81
Common bream	*Abramis brama*	12	2.03
Common bleak	*Alburnus alburnus*	12	0.29
European smelt	*Osmerus eperlanus*	9	0.19
Burbot	*Lota lota*	8	0.48
Tench	*Tinca tinca*	7	7.90
Vendace	*Coregonus albula*	7	1.07
Brown trout	*Salmo trutta*	7	1.06
Common whitefish	*Coregonus lavaretus*	6	2.19
Common minnow	*Phoxinus phoxinus*	4	0.02
European eel	*Anguilla anguilla*	3	0.39
Arctic char	*Salvelinus alpinus*	2	2.61
Carp species unidentified		2	0.05
Ide	*Leuciscus idus*	1	1.16
Zander/pikeperch	*Sander lucioperca*	1	0.51
Crucian carp	*Carassius carassius*	1	0.22
Alpine bullhead	*Cottus poecilopus*	1	1.16E-03
Ninespine stickleback	*Pungitius pungitius*	1	2.90E-04

Table S3 Lake-specific coefficients for the calculations of fish biomass production, based on length-at-age relationships $L = a * A^b$ (eq. 1), weight-at-length relationships $W = c * L^d$ (eq. 2), and weight-at-age relationships $W = f * A^g$ (eq. 3). Lake specific coefficients were also used for the size specific growth relationships $G_{L,t-1,lake} = \alpha_{lake} * e^{\beta_{lake} * L_{t-1}}$ (eq. 5). Some lakes do not have all values as we did not have all data for all lakes, see methods.

**Lake**	**Fish biomass production**						**Size specific growth**	
	a	b	c	d	f	g	α_{lake}	β_{lake}
Bäen	4.212	0.459	-12.001	3.112	1.109	1.427	4.923	-0.028
Örsjön	4.245	0.465	-12.021	3.109	1.176	1.446	12.949	-0.040
Brunnsjön	4.076	0.524	-11.975	3.097	0.648	1.621	6.001	-0.030
Stora Skärsjön	4.222	0.459	-11.876	3.085	1.147	1.416	13.681	-0.044
Gyltigesjön							7.071	-0.035
Harasjön	4.012	0.603	-12.040	3.114	0.455	1.878	2.445	-0.019
Hjärtsjön	4.015	0.918	-11.794	3.067	0.520	2.815	6.209	-0.018
Fiolen	4.022	0.755	-12.147	3.132	0.449	2.365	4.362	-0.018
Gyslättasjön	4.380	0.358	-11.544	3.006	1.621	1.076	13.477	-0.041
Nässjön	4.141	0.514	-11.822	3.061	0.856	1.574	5.540	-0.025
Hagsjön	3.735	0.879	-12.231	3.150	-0.467	2.769	3.877	-0.018
Hagasjön	4.053	0.693	-12.209	3.156	0.581	2.189	6.123	-0.020
Stengårdshultasjön	4.007	0.691	-12.167	3.127	0.360	2.162	4.133	-0.020
Stora Härsjön	4.026	0.736	-12.342	3.173	0.433	2.336	5.048	-0.020
Allgjutten	4.098	0.714	-12.099	3.107	0.634	2.219	5.441	-0.020
Fräcksjön	4.126	0.596	-12.133	3.128	0.776	1.866	5.692	-0.026
Granvattnet	4.038	0.613	-12.106	3.152	0.624	1.932	5.186	-0.024
Geten	4.128	0.477	-12.138	3.135	0.800	1.496	9.747	-0.039
Skärgölen							4.914	-0.022
Långsjön	3.996	0.669	-12.344	3.175	0.344	2.124	7.501	-0.031
Björken	4.061	0.701	-12.435	3.197	0.549	2.241	2.646	-0.015
Rotehogstjärnen	3.987	0.601	-12.190	3.141	0.334	1.889	6.488	-0.031
Ejgdesjön	4.244	0.748	-12.038	3.121	1.208	2.333	7.614	-0.019
Älgsjön	4.039	0.624	-12.097	3.128	0.537	1.952	4.272	-0.023
Stora Envättern	4.105	0.627	-12.297	3.165	0.693	1.984	4.874	-0.022
Västra Solsjön	3.989	0.873	-12.352	3.181	0.336	2.778	6.240	-0.018
Stensjön	3.885	0.754	-12.401	3.192	-0.001	2.408	3.781	-0.020
Långsjön	4.018	0.578	-12.369	3.178	0.401	1.836	5.865	-0.030
Årsjön	4.249	0.531	-12.153	3.131	1.153	1.661	8.491	-0.028
Bysjön							6.855	-0.026
Tärnan	4.097	0.612	-12.417	3.192	0.660	1.953	4.726	-0.024
Ulvsjön	4.120	0.589	-12.259	3.148	0.712	1.853	6.586	-0.029
Örvattnet	4.029	0.572	-12.130	3.128	0.475	1.788	6.153	-0.030
Lien	4.100	0.744	-12.341	3.164	0.629	2.355	6.495	-0.021
Övre Skärsjön	4.092	0.552	-11.997	3.088	0.642	1.706	4.554	-0.025
Dagarn	4.165	0.641	-12.422	3.180	0.822	2.038	5.669	-0.022
Västra Skälsjön	4.056	0.775	-12.312	3.155	0.484	2.445	9.152	-0.027

Skifsen	3.914	0.695	-12.167	3.124	0.062	2.172	4.641	-0.023
Tryssjön	4.344	0.484	-11.922	3.085	1.480	1.493	8.829	-0.029
Rädsjön	3.813	0.895	-12.106	3.122	-0.200	2.794	6.056	-0.024
Källsjön	3.958	0.589	-12.193	3.148	0.268	1.856	4.764	-0.028
Stensjön	4.113	0.547	-12.173	3.125	0.682	1.711	5.233	-0.026
Väster Rännöbodsjön	3.944	0.641	-12.364	3.176	0.164	2.035	4.170	-0.026
Stor-Backsjön	3.934	0.692	-12.418	3.185	0.114	2.204	2.472	-0.016
Degervattnet	4.021	0.687	-12.240	3.142	0.395	2.159	2.770	-0.016
Remmarsjön	3.944	0.672	-12.368	3.171	0.138	2.131	3.699	-0.021
Bjännsjön	3.920	0.700	-12.300	3.178	0.156	2.223	3.706	-0.021
Pahajärvi	4.013	0.612	-12.220	3.132	0.349	1.917	4.540	-0.024
Jutsajaure	3.957	0.686	-12.263	3.159	0.239	2.167	3.684	-0.020

Table S4 All models used in the model selection based on AICc, with explanatory variables T (mean temperature) and/or A (absorbance), excluding or including covariates D (mean depth) and P (total phosphorus). Bold are the best models (ΔAICc <2) within each 4-model-set (T, A, T+A, T*A; excluding [grey shaded] or including [white] covariates D and P), and their respective R^2 and adjusted R^2 values.

Response variable	Sign. In table 1	Explanatory variables	Covariates	AICc	ΔAICc	R^2	Adj R^2
**Fish biomass productivity** (LN) Nlakes = 46	T and A						
		T		115.96	4.81	0.12	0.10
		A		111.15	2.92	0.21	0.19
		**T + A**		**108.23**	**0**	**0.29**	**0.26**
		T*A		110.40	2.17	0.30	0.25
		T	D + P	115.14	7.94	0.22	0.17
		**A**	**D + P**	**108.36**	**1.16**	**0.33**	**0.28**
		**T + A**	**D + P**	**107.20**	**0**	**0.38**	**0.32**
		T*A	D + P	109.80	2.6	0.38	0.31
**Population biomass** (LN) Nlakes = 52	T						
		**T**		**121.82**	**0.7**	**0.11**	**0.10**
		A		124.78	3.66	0.06	0.04
		**T + A**		**121.12**	**0**	**0.16**	**0.13**
		T*A		123.53	2.41	0.16	0.11
		T	D + P	114.75	4.12	0.29	0.25
		A	D + P	112.95	2.32	0.32	0.28
		**T + A**	**D + P**	**110.63**	**0**	**0.38**	**0.33**
		**T*A**	**D + P**	**112.39**	**1.76**	**0.39**	**0.32**
**Population abundance** (LN) Nlakes = 52	T						
		**T**		**113.74**	**0**	**0.05**	**0.03**
		**A**		**114.96**	**1.22**	**0.02**	**0.00**
		**T + A**		**115.05**	**1.31**	**0.06**	**0.03**

		T*A		117.10	3.36	0.07	0.01
		T	D + P	98.80	8.98	0.35	0.31
		**A**	**D + P**	**89.83**	**0**	**0.45**	**0.42**
		**T + A**	**D + P**	**90.67**	**0.84**	**0.47**	**0.42**
		T*A	D + P	93.18	3.35	0.47	0.41
**Mean length** (LN)<br>Nlakes = 49	T						
		**T**		**-47.79**	**0.82**	**0.16**	**0.15**
		A		-40.96	7.65	0.04	0.02
		**T + A**		**-46.89**	**1.72**	**0.19**	**0.15**
		**T*A**		**-48.61**	**0**	**0.26**	**0.21**
		**T**	**D + P**	**-48.98**	**0**	**0.26**	**0.21**
		A	D + P	-40.59	8.39	0.12	0.06
		T + A	D + P	-46.53	2.45	0.26	0.20
		T*A	D + P	-46.90	2.08	0.31	0.23
**Skewness (positive) in length**<br>Nlakes = 49	T						
		**T**		**114.00**	**0**	**0.27**	**0.26**
		A		129.14	15.14	0.01	-0.01
		T + A		116.29	2.29	0.27	0.24
		T*A		117.93	3.93	0.29	0.24
		**T**	**D + P**	**114.81**	**0**	**0.33**	**0.28**
		A	D + P	129.15	14.34	0.10	0.04
		**T + A**	**D + P**	**115.88**	**1.07**	**0.35**	**0.29**
		T*A	D + P	118.31	3.5	0.35	0.28
**Mean predicted age** (LN)<br>Nlakes = 46	T and A						
		T		-12.72	2.92	0.22	0.20
		A		-3.58	12.06	0.04	0.02
		**T + A**		**-14.64**	**1**	**0.29**	**0.25**
		**T*A**		**-15.64**	**0**	**0.34**	**0.29**
		T	D + P	-8.64	2.55	0.23	0.18
		A	D + P	-1.95	9.24	0.11	0.05
		**T + A**	**D + P**	**-11.03**	**0.16**	**0.31**	**0.24**
		**T*A**	**D + P**	**-11.19**	**0**	**0.35**	**0.27**
**Skewness (positive) in age**<br>Nlakes = 42	T						
		**T**		**123.33**	**0**	**0.29**	**0.27**
		A		137.69	14.36	0.00	-0.02
		T + A		125.77	2.44	0.29	0.25
		T*A		128.24	4.91	0.29	0.24
		**T**	**D + P**	**126.53**	**0**	**0.32**	**0.27**
		A	D + P	139.18	12.65	0.08	0.01
		**T + A**	**D + P**	**127.77**	**1.24**	**0.34**	**0.27**
		T*A	D + P	130.42	3.89	0.35	0.26
**Length-at-age 1** (LN)<br>Nlakes = 49	T and A						
		T		-80.46	5	0.07	0.05
		A		-82.95	2.51	0.12	0.10
		**T + A**		**-85.46**	**0**	**0.20**	**0.17**
		**T*A**		**-84.83**	**0.63**	**0.23**	**0.18**
		**T**	**D + P**	**-79.09**	**1.67**	**0.13**	**0.08**
		**A**	**D + P**	**-78.82**	**1.94**	**0.13**	**0.07**
		**T + A**	**D + P**	**-80.76**	**0**	**0.21**	**0.13**

		**T*A**	**D + P**	**-79.64**	**1.12**	**0.24**	**0.14**
**Length-at-age 6** (LN)<br>Nlakes = 49	A						
		T		-14.01	23.36	0.00	-0.02
		**A**		**-37.37**	**0**	**0.39**	**0.37**
		T + A		-35.04	2.33	0.39	0.36
		T*A		-33.09	4.28	0.39	0.35
		T	D + P	-25.75	10.4	0.29	0.24
		**A**	**D + P**	**-36.15**	**0**	**0.43**	**0.39**
		T + A	D + P	-33.55	2.6	0.43	0.38
		T*A	D + P	-32.58	3.57	0.45	0.39
**Length-specific-growth rate 170 mm** (LN)<br>Nlakes = 49	A						
		T		129.05	6.16	0.02	0.00
		**A**		**122.89**	**0**	**0.13**	**0.12**
		**T + A**		**124.62**	**1.73**	**0.15**	**0.11**
		T*A		127.09	4.2	0.15	0.09
		T	D + P	128.48	2.33	0.12	0.06
		**A**	**D + P**	**126.15**	**0**	**0.16**	**0.11**
		**T + A**	**D + P**	**127.83**	**1.68**	**0.18**	**0.10**
		T*A	D + P	130.54	4.39	0.18	0.08

Table S5 All models used in the model selection based on AICc, with explanatory variables T (mean temperature) and/or A (absorbance), excluding or including the covariate roach population biomass (R). Bold are the best models (ΔAICc <2) within each 4-model-set (T, A, T+A, T*A; excluding [grey shaded] or including [white] the covariate R), and their respective R^2 and adjusted R^2 values.

**Response variable**	**Sign. In table 1**	**Explanatory variables**	**Covariates**	**AICc**	**ΔAICc**	**R^2**	**Adj R^2**
**Fish biomass productivity** (LN)<br>Nlakes = 46	T and A						
		T		115.96	4.81	0.12	0.1
		A		111.15	2.92	0.21	0.19
		**T + A**		**108.23**	**0**	**0.29**	**0.26**
		T*A		110.40	2.17	0.30	0.25
		T	R	111.93	7.23	0.23	0.20
		A	R	108.75	4.05	0.28	0.25
		**T + A**	**R**	**104.70**	**0.00**	**0.38**	**0.34**
		T*A	R	107.04	2.34	0.38	0.32
**Population biomass** (LN)<br>Nlakes = 52	T						
		**T**		**121.82**	**0.7**	**0.11**	**0.1**
		A		124.78	3.66	0.06	0.04
		**T + A**		**121.12**	**0**	**0.16**	**0.13**
		T*A		123.53	2.41	0.16	0.11
		**T**	**R**	**114.84**	**0.00**	**0.26**	**0.23**
		A	R	120.66	5.82	0.17	0.14
		**T + A**	**R**	**115.44**	**0.60**	**0.28**	**0.24**
		T*A	R	117.86	3.02	0.29	0.23

**Population abundance** (LN) Nlakes = 52	T						
		**T**		**113.74**	**0**	**0.05**	**0.03**
		**A**		**114.96**	**1.22**	**0.02**	**0**
		**T + A**		**115.05**	**1.31**	**0.06**	**0.03**
		T*A		117.1	3.36	0.07	0.01
		**T**	**R**	**99.64**	**0.00**	**0.30**	**0.28**
		A	R	103.23	3.59	0.25	0.22
		T + A	R	101.91	2.27	0.31	0.26
		T*A	R	104.21	4.56	0.31	0.25
**Mean length** (LN) Nlakes = 49	T						
		**T**		**-47.79**	**0.82**	**0.16**	**0.15**
		A		-40.96	7.65	0.04	0.02
		**T + A**		**-46.89**	**1.72**	**0.19**	**0.15**
		**T*A**		**-48.61**	**0**	**0.26**	**0.21**
		**T**	**R**	**-46.17**	**0.89**	**0.18**	**0.14**
		A	R	-39.88	7.18	0.06	0.02
		**T + A**	**R**	**-45.58**	**1.48**	**0.21**	**0.16**
		**T*A**	**R**	**-47.06**	**0.00**	**0.27**	**0.21**
**Skewness (positive) in length** Nlakes = 49	T						
		**T**		**114.00**	**0**	**0.27**	**0.26**
		A		129.14	15.14	0.01	-0.01
		T + A		116.29	2.29	0.27	0.24
		T*A		117.93	3.93	0.29	0.24
		**T**	**R**	**115.40**	**0.00**	**0.29**	**0.25**
		A	R	130.33	14.93	0.03	-0.01
		T + A	R	117.68	2.28	0.29	0.24
		T*A	R	119.51	4.11	0.30	0.24
**Mean predicted age** (LN) Nlakes = 46	T and A						
		T		-12.72	2.92	0.22	0.2
		A		-3.58	12.06	0.04	0.02
		**T + A**		**-14.64**	**1**	**0.29**	**0.25**
		**T*A**		**-15.64**	**0**	**0.34**	**0.29**
		T	R	-10.81	3.33	0.23	0.19
		A	R	-1.63	12.51	0.05	0.01
		**T + A**	**R**	**-13.08**	**1.07**	**0.30**	**0.25**
		**T*A**	**R**	**-14.15**	**0.00**	**0.36**	**0.29**
**Skewness (positive) in age** Nlakes = 42	T						
		**T**		**173.42**	**0**	**0.29**	**0.27**
		A		180.56	14.36	0.00	-0.02
		T + A		175.40	2.44	**0.29**	0.25
		T*A		177.80	4.91	0.29	0.24
		**T**	**R**	**82.58**	**0.00**	**0.25**	**0.21**
		A	R	92.46	9.87	0.05	0.00
		T + A	R	84.77	2.19	0.25	0.19
		T*A	R	87.27	4.69	0.26	0.18
**Length-at-age 1** (LN) Nlakes = 49	T and A						
		T		-80.46	5	0.07	0.05
		A		-82.95	2.51	0.12	0.1

		**T + A**		**-85.46**	**0**	**0.20**	**0.17**
		**T*A**		**-84.83**	**0.63**	**0.23**	**0.18**
		T	R	-80.38	7.34	0.11	0.07
		A	R	-83.16	4.56	0.16	0.13
		**T + A**	**R**	**-87.72**	**0.00**	**0.28**	**0.23**
		**T*A**	**R**	**-86.41**	**1.31**	**0.30**	**0.23**
**Length-at-age 6** (LN)<br>Nlakes = 49	A						
		T		-14.01	23.36	0.00	-0.02
		**A**		**-37.37**	**0**	**0.39**	**0.37**
		T + A		-35.04	2.33	0.39	0.36
		T*A		-33.09	4.28	0.39	0.35
		T	R	-13.15	27.00	0.03	-0.01
		**A**	**R**	**-40.14**	**0.00**	**0.45**	**0.42**
		T + A	R	-37.82	2.33	0.45	0.41
		T*A	R	-35.92	4.23	0.46	0.41
**Length-specific-growth rate 170 mm** (LN)<br>Nlakes = 49	A						
		T		129.05	6.16	0.02	0
		**A**		**122.89**	**0**	**0.13**	**0.12**
		**T + A**		**124.62**	**1.73**	**0.15**	**0.11**
		T*A		127.09	4.2	0.15	0.09
		T	R	131.23	6.76	0.02	-0.02
		**A**	**R**	**124.46**	**0.00**	**0.15**	**0.11**
		**T + A**	**R**	**126.36**	**1.90**	**0.16**	**0.10**
		T*A	R	128.95	4.49	0.16	0.08
